# The Therapeutic Value of the Iodide of Potassium

**Published:** 1875-03

**Authors:** 


### Miscellaneous.

### The Therapeutic Value of Iodide of Potassium.

As I have for several years paid considerable attention to the action of iodide of potassium, I venture to offer the following remarks as supplementary to Mr. Spurgin's article in the *Journal* of September 5th, 1874. This medicine has been accredited with many modes of action: thus, in struma as an alterative, in asthma as a sedative, and in diphtheria as an antidote. To all these titles it may have a claim which different observers may think fairly borne out; but certainly the one distinct and indisputable action of iodide of potassium which I have noticed, is that of *stimulating the mucus membranes;* thereby influencing their action and promoting their secretions. Thus, as the results of its use, there are pain and sense of fulness across the eyes; increased secretion from the nares, mouth, fauces, and bronchi, leucorrhea and menorrhagia are greatly aggrevated; and in persons very susceptible of its influence, diarrhœa is induced, not so much of cathartic as of a dysenteric kind; that is, rather an increase of mucus with tenesmus than of serum with catharsis.

In a person suffering from an attack of chronic winter cough, the first symptoms are great difficulty in breathing, amounting to a sense of suffocation; hard, dry, racking cough, which the patient says he cannot subdue; while he expresses a belief that relief would be obtained if something could be brought up The suffocation complained of has been attributed to a swollen state of the air-passages, obstructing the respiration; but there is a fair probability that the dry congested condition of the membrane is unfavorable to the interchange of gases requisite for blood aeration, and the situation of the patient such that, however he may fill his lungs, his sufferings remains unrelieved. Whatever the actual state of matters at this point, certain it is, that as soon as expectoration sets in, the breathing is improved; and, although the disease has by no means gone, the patient is so far better. Many hours of severe suffering may be obviated by taking advantage of the power of iodide of potassium to restore and promote the secretion of the bronchial membranes, thereby greatly relieving the congested blood-vessels, producing comparative tranquility of breathing, and getting the patient over the first stage of the disease much sooner than he otherwise would. This, however, is possibly not its only value. For, here again, however opinions may differ as to the cause of the emphysema, which from an early period exists in these cases, no one can have witnessed the severe and straining cough at the onset of the attack, without feeling that it is at least possible for either dilatations of the air-cells or rupture of the tissue of the lung to take place—complications much less likely to occur, so far as the cough is concerned, when the sputum has been rendered easier of expectoration, and the irritability of the congested membranes removed by free secretion. It is further to be remarked that the action of the iodide of potassium changes the purulent character of the sputa in chronic bronchitis to a much healthier appearance. From this view of its operation, it follows as a matter of course, that when free secretion of mucus has set in the medicine should be used with caution or altogether abandoned; and, therefore, when in the treatment of bronchitis—capillary or chronic—moist *râles* are fairly established, the further management of the case should be on the principle of preventing a too abundant secretion, at the same time employing such means as may assist expectoration and maintain the strength.

In asthma, iodide of potassium is recognized as a valuable medicine. Here the explanation of its action generally given, is that of a sedative relieving bronchial spasm; evidence of the presence of spasm being found in the wheezing and whistling sounds heard in ausculation. Either of these sounds, however, fairly suggests the question, how far a fit of asthma is dependent on, or, at all events, greatly aggravated by, an abnormally dry condition of the mucous membranes, acting as in the diseases already mentioned, which is relieved by the iodide restoring the secretion.

In diphtheria, iodide of potassium is looked upon by many practitioners as the best remedy we possess. Here its alterative and sedative actions are laid aside, and we have it doing duty as an antidote to the diphtheric poison; although, so far as can be seen, it exercises no new influence. In this disease, while there is free secretion from the nares, the breathing and cough sounds are usually not very alarming, nor is respiration greatly impeded. It is not till the nares become dry—and doubtless the pharyngeal, laryngeal, and tracheal secretions diminish—that the formation of false membrane proceeds with fatal rapidity; hence, it does not seem too much to assume, so long as an iodide can keep up these secretions in such profusion as to prevent them from remaining on the parts sufficiently long to undergo membranous change, so long will its action be beneficial. The idea of an antidote might be more satisfactory; but it cannot be substantiated; nor does this view of its action afford any indication as to what extent the medicine should be given: whereas, by paying attention to the degree of influence exerted upon the mucous secretions, the dose and frequency of administration may fairly be ascertained; if not, indeed, the knowledge acquired as to whether or not it is doing any good.

Without at present entering into a consideration of the influence of iodide of potassium on digestion and assimilation—the real sources of its so-called alterative power—I may state as my conviction, that in all the various manifestations of struma, etc., where this medicine is of service it acts, so far as the iodide is concerned, in stimulating the mucous membrane of the stomach and duodenum—possibly, by sympathetic action, the liver and pancreas also —to increase secretion, whilst its alkaline base tends to promote the digestion of fat and starch.

For the dose no absolute rule can be laid down, because, in few respects, indeed, do constitutions and temperaments differ more than in the relative irritability of the mucous membranes, and, consequently, the power of iodine to influence their action. Persons of the bilious temperament usually resist its power to a wonderful degree, whilst in those of the lymphatic, sanguineous, and above all, the nervous, a few doses of two grains each will often suffice to cause coryza, ptyalism, pharyngeal irritation, and cough. In such diseases as diphtheria, the object should be to produce its influence as rapidly as possible, whilst in others, as struma, small doses long continued are preferable.—James Lawrie, M. D., Glasgow.—*The British Medical Journal.—New Orleans Medical and Surgical Journal.*

